# Cannabinoid Signaling in Auditory Function and Development

**DOI:** 10.3389/fnmol.2021.678510

**Published:** 2021-05-17

**Authors:** Sumana Ghosh, Kendra Stansak, Bradley J. Walters

**Affiliations:** ^1^Department of Neurobiology and Anatomical Sciences, University of Mississippi Medical Center, Jackson, MS, United States; ^2^Department of Otolaryngology—Head and Neck Surgery, University of Mississippi Medical Center, Jackson, MS, United States

**Keywords:** cannabinoid, cochlea, spiral ganglion, hearing, hearing—drug effects, otoprotection, hair cell

## Abstract

Plants of the genus *Cannabis* have been used by humans for millennia for a variety of purposes. Perhaps most notable is the use of certain *Cannabis* strains for their psychoactive effects. More recently, several biologically active molecules within the plants of these *Cannabis* strains, called phytocannabinoids or simply cannabinoids, have been identified. Furthermore, within human cells, endogenous cannabinoids, or endocannabinoids, as well as the receptors and secondary messengers that give rise to their neuromodulatory effects, have also been characterized. This endocannabinoid system (ECS) is composed of two primary ligands—anandamide and 2-arachidonyl glycerol; two primary receptors—cannabinoid receptors 1 and 2; and several enzymes involved in biosynthesis and degradation of endocannabinoid ligands including diacylglycerol lipase (DAGL) and monoacylglycerol lipase (MAGL). Here we briefly summarize cannabinoid signaling and review what has been discerned to date with regard to cannabinoid signaling in the auditory system and its roles in normal physiological function as well as pathological conditions. While much has been uncovered regarding cannabinoid signaling in the central nervous system, less attention has been paid to the auditory system specifically. Still, evidence is emerging to suggest that cannabinoid signaling is critical for the development, maturation, function, and survival of cochlear hair cells (HCs) and spiral ganglion neurons (SGNs). Furthermore, cannabinoid signaling can have profound effects on synaptic connectivity in CNS structures related to auditory processing. While clinical cases demonstrate that endogenous and exogenous cannabinoids impact auditory function, this review highlights several areas, such as SGN development, where more research is warranted.

## Introduction

Humans have used products derived from *Cannabis* plants for millennia. Traditional uses of these plants include the derivation of fibers for textiles, rope, and article, and the use of other parts of the plants as a source of food, for medicinal purposes, and for psychoactive effects in spiritual or recreational use (Ren et al., 2019). Though there is some debate of the exact taxonomy, as many as four different strains or varieties of *Cannabis* have been classified, including *Cannabis sativa*, *C. indica*, *C. ruderalis*, and *C. afghanica* (Clarke and Merlin, 2013; Small, 2015). All four strains can contain psychoactive compounds. However, these designations may not be overly informative with regard to the amounts of bioactive molecules as natural and artificial selection have led to vast differences within and across strains (Clarke and Merlin, 2013). In the past few decades, extensive research has focused on isolating and characterizing cannabinoid compounds, as well as their receptors and biological functions. Originally the term “cannabinoids” referred to more than 60 C21 terpenophenolic compounds produced by *Cannabis* plants. Δ9-tetrahydrocannabinol (THC), the major psychoactive compound produced by this plant, was first isolated and characterized by Mechoulam and Gaoni (1967). Currently, there are 120 pharmacologically distinct compounds identified as being produced by these plants which are broadly known as phytocannabinoids (Reekie et al., 2018). Some of the other important phytocannabinoids include cannabidiol (CBD), cannabinol (CBN), cannabichromene (CBC), and cannabigerol (CBG; Morales et al., 2017). Shortly after the discovery of phytocannabinoids, Howlett et al. provided compelling evidence that pharmacological activities of phytocannabinoids are modulated by G-protein coupled receptors (GPCRs) which were later cloned and characterized as cannabinoid receptor 1 (CB1) and cannabinoid receptor 2 (CB2; Howlett and Fleming, 1984; Matsuda et al., 1990; Munro et al., 1993; Howlett et al., 2002). The endogenous cannabinoid ligands or endocannabinoids (ECBs) are lipophilic molecules that are synthesized as a product of arachidonic acid metabolism. The principal components of the endocannabinoid system (ECS) include: (1) the ECB ligands, (2) the enzymes involved in the biosynthesis and degradation of ECB ligands, (3) the ECB receptors, and (4) the ECB membrane transporter proteins (EMTs).

Despite the rapidly growing body of information pertaining to the physiological roles of the ECS in different tissues including the brain, significantly less is known about the distribution and function of the ECS in the peripheral and central tissues of the auditory system. Here we will review the different components of ECB signaling in general and what is currently known about the physiological and pathophysiological role of this system in the auditory circuit. Furthermore, this review will consider hypotheses of putative roles the ECS may have in cochlear development and maturation, based on potentially analogous roles of ECB signaling in other tissues.

## The Endocannabinoid System: A Classical View

### Biosynthesis, Transportation, and Degradation of Endocannabinoids (ECBs)

**Figure 1:**
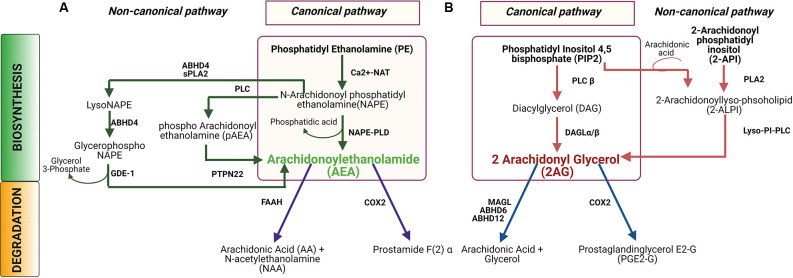
Biosynthesis and degradation of N-arachidonoyl ethanolamine (AEA) and 2-arachidonoyl glycerol (2AG). **(A)** In the canonical pathway for AEA synthesis and catabolism, an N-acetyl group is added to phosphatidyl ethanolamine (PE) to produce N-arachidonoyl PE, or NAPE, which is then converted to AEA by the enzyme NAPE-PLD. In a non-canonical pathway, NAPE can be hydrolyzed by PLC to produce pAEA, and subsequently dephosphorylated by PTPN22N to produce AEA. Alternatively, NAPE can be converted to lyso-NAPE by the catalytic activity of either ABHD4 or sPLA2, and subsequently, lyso-NAPE is hydrolyzed by ABHD4 and GDE-1 to produce AEA. AEA is hydrolyzed by fatty acid amide hydrolase (FAAH) to produce AA and NAA or, AEA can be oxidized by COX2 to produce prostamide F(2)α. **(B)** In the canonical biosynthesis pathway for 2AG, PIP2 is hydrolyzed by PLCβ to DAG which is subsequently hydrolyzed by DAGL to produce AG. In the non-canonical pathway, 2-ALPI is synthesized from either PIP2 or 2-API, which is subsequently hydrolyzed by lyso-PI-PLC to generate 2-AG. Degradation of 2AG occurs by hydrolysis by either MAGL or ABHD6 or ABHD12 to produce arachidonic acid and glycerol. Alternatively, 2AG can be oxidized by COX2 to generate to PGE2-G. Abbreviations: NAT, N-acetyltransferase; NAPE-PLD, N-acyl phosphatidylethanolamine-specific phospholipase D; PLC, Phospholipase C; PTPN22, Protein tyrosine phosphatase, non-receptor type 22 (lymphoid); ABHD4, α/β-Hydrolase domain containing 4; sPLA2, Secretory phospholipases A2; GDE-1, Glycerophosphodiester phosphodiesterase-1; FAAH, Fatty acid amide hydrolase; COX2, Cycloxygenase2; DAGL, Diacylglycerol lipase; Lyso-PI-PLC, Lyso phosphatidyl inositol phospholipase C; MAGL, Monoacylglycerol lipase; ABHD6, α/β-Hydrolase domain containing 6; ABHD12, α/β-Hydrolase domain containing 12.

Most of our knowledge of ECB synthesis comes from studies in the central nervous system (CNS) of rodent models, where the two main ECBs are anandamide (N-arachidonoyl ethanolamine, or AEA) and 2-arachidonoyl glycerol (2AG). AEA and 2AG are synthesized in a spatiotemporally regulated manner “on-demand” from their phospholipid precursor molecules present in the plasma membrane (Di Marzo et al., 1999; Kim et al., 2002). According to this classical view, depolarization or G-protein coupled signaling in neural cells can induce ECB production from lipid precursors *via* activation of the calcium-dependent enzymes involved in their biosynthesis (Basavarajappa, 2007a; Nyilas et al., 2008). In the canonical pathway (Figure 1A), AEA is synthesized from the precursor molecule n-arachidonylphosphatidylethanolamine (NAPE) by catalytic actions of N-acetyl transferase and phospholipase D (Di Marzo et al., 1994; Kim et al., 2002; Lu and MacKie, 2016). Even though AEA and 2AG share a common arachidonic acid backbone, 2AG is generated by a different two-step process (Figure 1B) beginning with phosphatidylinositol-4, 5-bisphosphate (PIP2). First, PIP2 is cleaved by phospholipase C (β or γ) to generate inositol-1,4,5 triphosphate, and diacyl glycerol (DAG). DAG is then hydrolyzed by serine hydrolase diacylglycerol lipase (DAGL) α or β to produce 2AG (Baggelaar et al., 2018). Once released into the intercellular space, ECBs can act at adjacent cell membranes *via* their receptors, or possibly *via* other, receptor-independent mechanisms (Chicca et al., 2012). Upon being transported intracellularly, both AEA and 2AG have very short half-lives and are almost instantly catabolized to arachidonic acid and ethanolamine or arachidonic acid and glycerol, respectively (Deutsch and Chin, 1993; Dinh et al., 2002). AEA is primarily degraded by fatty acid amide hydrolase (FAAH) in the central nervous system (CNS) (Lu and MacKie, 2016) to produce arachidonic acid and ethanolamine. Alternatively, AEA can either be oxidized by cyclooxygenase-2 (COX2) to produce prostamide (Woodward et al., 2008) or be hydrolyzed by N-acylethanolamine-hydrolyzing acid amidase (NAAA) (Tsuboi et al., 2005). In the CNS, 2AG can be hydrolyzed by four different enzymes namely monoacylglycerol lipase (MAGL), which accounts for the majority of 2AG hydrolysis, but also α/β-Hydrolase domain containing 6 (ABHD6), α/β-Hydrolase domain containing 12 (ABHD12), or COX2. These enzymes are located in different cellular and subcellular compartments. MAGL is a soluble enzyme, distributed at the presynaptic terminal, while ABHD6 and ABHD12 are integrated in the membrane. ABHD6 is primarily found in neuronal dendrites whereas ABHD12 is abundantly expressed in microglia (Cravatt et al., 2001; Blankman et al., 2007; Kano et al., 2009; Marrs et al., 2010). 2AG can also be oxidized by COX2 to produce prostaglandin E2 glycerol ester (PGE2-GE) which has been implicated in the modulation of synaptic transmission and synaptic plasticity in the hippocampus (Sang et al., 2006; Yang et al., 2008; Urquhart et al., 2015). For more detailed information on ECB metabolism, transport and signaling, readers are referred to the review by Lu and MacKie (2016).

### Cannabinoid Receptors and Signaling Pathways

According to the classical model, all cannabinoids, including endogenous, synthetic, and phyto-cannabinoids, mediate their actions by binding to two types of receptors- cannabinoid receptors and non-cannabinoid receptors. There are primarily two types of cannabinoid receptors, the CB1 and CB2 receptors, which are encoded by the *CNR* genes, *CNR1* and *CNR2* respectively. At the protein level, the human CB1 receptor shares 44% sequence homology with the CB2 receptor. In terms of conservation across species, CB1 receptors are highly similar in rodents and humans (97–99%) compared to the CB2 receptor which is less well conserved (~80%; Goedert et al., 1998; Buckley, 2008; Liu et al., 2009; Zhang et al., 2015). Differential isoforms or splice variants of both of the receptors can be distinctly expressed across various tissues (Ryberg et al., 2005; Liu et al., 2009; González-Mariscal et al., 2016). The CB1 receptor is abundantly expressed in the CNS where it acts largely as a neuromodulator (Lovinger, 2008). These CB1 receptors are predominantly found at presynaptic inhibitory GABAergic terminals and to a lower extent at the presynaptic terminals of excitatory glutamatergic and dopaminergic neurons. CB1 receptors can also be found in postsynaptic terminals in the cerebral cortex, and in glial cells (Salio et al., 2002; Bacci et al., 2004; Kushmerick et al., 2004; Domenici et al., 2006; Degroot et al., 2006). Outside of the nervous system, CB1 receptors have also been detected in cardiac tissues, ovaries, adrenal glands, and immune cells (Cecconi et al., 2014; Maccarrone et al., 2015; Hillard et al., 2016). Somewhat in contrast to CB1 receptors, CB2 receptors are predominantly found in immune cells including CNS microglia and in numerous cells of the immune system and the gastrointestinal tract. Accordingly, CB2 signaling has been shown to regulate immune cell survival, cytokine production, and stress response and thus it acts as a major immunomodulator (Basu and Dittel, 2011). In addition to the cannabinoid receptors, multiple non-CB1/CB2 receptors have also been identified in recent decades as potential mediators of cannabinoid signaling, including capsaicin-sensitive transient receptor potential (TRP) channels, including TRP vanilloid 1 (TRPV1) and other members of the TRPV family. Orphan G-protein coupled receptor 55 (GPR 55) and nuclear receptor peroxisome-proliferator-activated receptors (PPAR) have also been identified as receptors that can be bound by cannabinoids and initiate downstream signaling cascades (Sun and Bennett, 2007; Muller et al., 2009).

**Figure 2:**
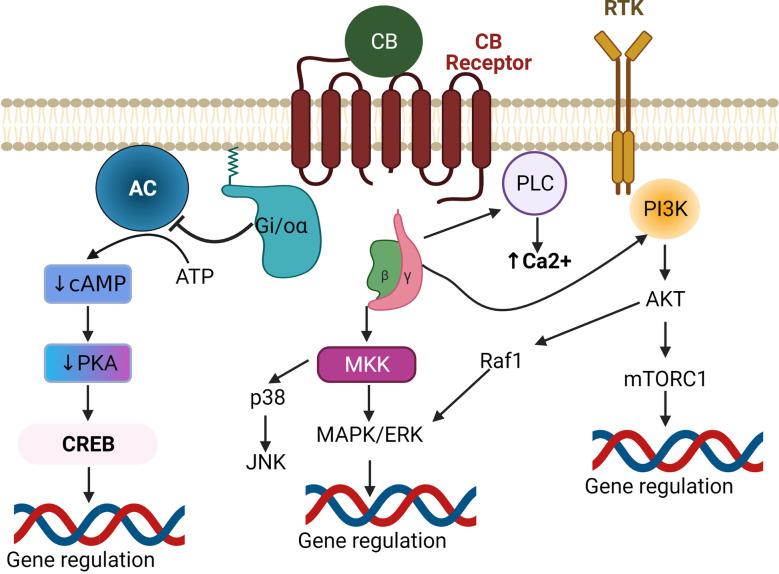
Canonical endocannabinoid signaling pathways. Upon binding of a CB ligand to a Gi/o-coupled GPCR, the Gi/o-α receptor subunit gets detached from the βγ subunits. The liberated Gi/o-α inhibits adenylate cyclase (AC) causing subsequent decreases in cAMP-mediated PKA and CREB activation, leading to downregulation of CREB-induced gene expression. The βγ subunits activate either MAPK or PI3K to regulate gene expression. Alternatively, they can also regulate Ca^2+^ levels *via* activation of PLC. Inhibition of PKA can also affect other MAPK pathways (not shown). Abbreviations: GPCR, G protein-coupled receptors; AC, Adenylate Cyclase; cAMP, Cyclic adenosine monophosphate; PKA, Protein Kinase A; CREB, cAMP response element-binding protein; MKK, MAP kinase kinase; MAPK, Mitogen-activated protein kinase; ERK, Extracellular signal-related kinase; JNK, c-Jun N-terminal kinase; Raf1, Rapidly Accelerated Fibrosarcoma1; PI3K, Phosphoinositide 3-kinases; Akt, Protein kinase B; mTORC1, mammalian target of rapamycin complex 1; PLC, Phospholipase C.

**Figure 3:**
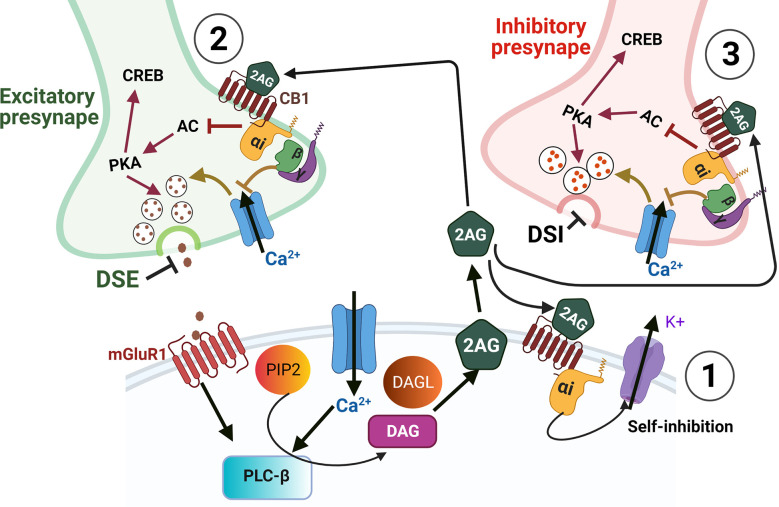
Endocannabinoid-mediated synaptic suppression. Depolarization and/or calcium influx into a postsynaptic cell leads to endocannabinoid synthesis. ECS ligands are then released into the extracellular space where they can bind cell autonomously to membrane-bound CB receptors **(1)**, or to receptors on presynaptic cells **(2,3)**, or to receptors on glial processes (not shown). Cell autonomous binding of ECS ligands **(1)** can modulate the strength of post-synaptic responses by self-inhibition *via* potassium efflux. In presynaptic terminals **(2,3)** ECS ligands bind to CB receptors which are Gi/o-coupled GPCRs. The binding of these GPCRs leads to Gi/o-α receptor subunit detachment from βγ subunits. The detached Gi/o-α then inhibits adenylate cyclase (AC), which would otherwise drive cAMP-mediated activation of PKA and CREB. However, with AC inhibited the decreased activation of PKA results in decreased vesicle release of neurotransmitters. The presynaptic release is also inhibited by the βγ subunits which inhibit Ca^2+^ entry that would also otherwise drive neurotransmitter release. When inhibitory neurotransmitters are prevented from being released by ECS it is termed depolarization-induced suppression of inhibition (DSI) and when ECS acts on excitatory presynaptic boutons, the inhibition of vesicle release is termed depolarization-induced suppression of excitation (DSE). In addition to DSI and DSE, the inhibition of AC and PKA can lead to decreased activation of CREB thus altering the expression of CREB dependent genes. Furthermore, the GPCR subunits and their effects on PKA and PKC can also activate either MAPK or PI3K signaling cascades to regulate gene expression (see Figure 2).

In terms of the ECB ligands, 2AG levels tend to be much higher than AEA in the CNS, and thus 2AG is suspected to be the primary endogenous ligand in nervous tissues. Elevated Ca^2 + ^ or activated Gq/11 coupled GPCR triggers the release of 2AG from the postsynaptic terminal, where it then traverses the synaptic cleft and binds to CB receptors at the presynaptic terminal (Castillo et al., 2012; Zou and Kumar, 2018). Both CB1 and CB2 receptors mediate their biological effects *via* pertussis toxin-sensitive G protein-coupled receptors (Figure 2) G_i_ and G_o_ (Howlett et al., 2002). Upon ligand binding, the G_i/o_ α subunit gets dissociated from G_i/o_ βγ. The GTP-bound α subunit reduces the activity of cAMP and protein kinase A *via* inhibition of adenylate cyclase. Meanwhile, the Gβγ subunit inhibits Ca^2+^ influx through voltage-gated Ca^2+^ channels (Wilson et al., 2001) and activates inwardly rectifying potassium channels (Figure 3), in a cell-dependent manner (Howlett et al., 2002). Additionally, ligand binding to CB1 or CB2 receptors can activate mitogen-activated protein kinases (MAPK) including c-Jun NH_2_-terminal kinases (JNK), extracellular signal related kinase (ERK), and p38 (Figure 2). Upon ligand binding, CB1 receptors can also induce PI3K/Akt activation in many cell types including glial cells where it promotes cell survival (Galve-Roperh et al., 2002; Gomez et al., 2011). The ECB mediated retrograde signaling can regulate short-term synaptic plasticity (Figure 3) *via* depolarization-induced suppression of inhibition (DSI) or depolarization-induced suppression of excitation (DSE). ECB signaling can also mediate long-term synaptic plasticity by either long-term depression (LTD) or long-term potentiation (LTP) (Marsicano et al., 2002; Basavarajappa, 2007b). Furthermore, AEA can also induce LTD by binding to the non-CB receptor TRPV1 thereby decreasing glutamate signaling (Grueter et al., 2010) and negatively regulating 2AG synthesis and 2AG-mediated signaling (Maccarrone et al., 2008). Modulation of synaptic signaling in these ways is well-known to affect motor behavior, anxiety, and memory formation but is likely equally important for sound sensation, perception, and comprehension.

## Endocannabinoids in The Auditory System

The mammalian auditory system collects sound energy *via* the external ear, where it is subsequently transitioned through solid and then liquid media in the middle and then inner ear. Within the inner ear, the mechanical energy of the acoustic stimulus displaces the basilar membrane of the cochlea causing the stereocilia of mechanosensory hair cells to be deflected against the tectorial membrane. The deflection of the stereocilia leads to increased opening of mechanoelectrotransduction channels thus converting the sound stimuli to electrical depolarization and subsequent chemical neurotransmission from cochlear hair cells in the inner ear to the cochlear nucleus (CN) of the brainstem. Afferent sensory input is then transmitted from the CN to the auditory cortex *via* the superior olivary complex (SOC), lateral lemniscus (LL), inferior colliculus (IC), and medial geniculate body (MGB). The ECB system is distributed throughout this entire auditory circuit where it has been shown to influence various glycinergic, glutaminergic (Kushmerick et al., 2004; Zhao et al., 2009), and cholinergic signals (Kushmerick et al., 2004; Zhao and Tzounopoulos, 2011) to modulate auditory function and perception.

### CB Signaling in the Cochlea and SGNs

The organ of Corti within the cochlea of the inner ear is comprised of two types of sensory hair cells (HCs), inner hair cells (IHC) and outer hair cells (OHC), and also non-sensory cells called supporting cells (SCs). Critical to cochlear function are a number of additional cell types that line the cochlear duct and perform numerous mechanical and physiological functions including maintenance of the endocochlear potential. Sound is transduced by the HCs, and in particular the IHCs which, upon depolarization, release glutamate which binds to postsynaptic receptors at the primary afferents of SGNs.

**Figure 4:**
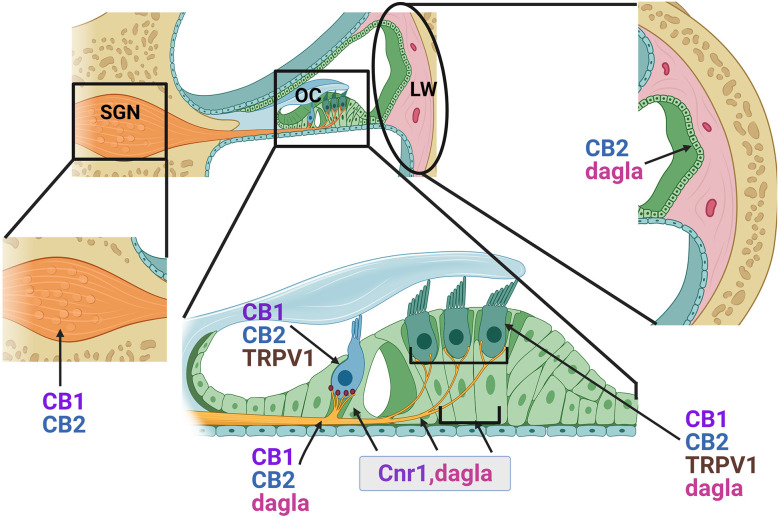
Distribution of endocannabinoid components in the adult cochlea. Based on immunohistochemical data and gene expression studies from previously published reports, CB1 and CB2 receptors (proteins) and CB1 transcripts (*Cnr1*) are distributed in the outer hair cells (OHC), inner hair cells (IHC), lateral wall (LW) cells and spiral ganglion neurons (SGN, cell bodies, and axonal projections). *Cnr1* transcripts were also found in the pillar cells (PC) and Dieter’s cells (DC). While CB2 receptors appear fairly widely distributed, immunolabeling suggested possible greater intensities of staining around the base of the IHCs where the SGN nerve fibers and the IHC ribbon synapses are located. Transcripts of D*agla* are expressed in the sensory HCs, PCs, Deiters cells (DC), and SGN. TRPV1 is expressed in both inner and outer HCs.

A number of studies over the past 15 years have demonstrated the expression of several ECS components in the mammalian cochlea (Figure 4). A preliminary study by Fauser and colleagues first provided evidence of presynaptic expression of CB1 receptors in both type I and type II SGNs in the adult gerbil cochlea. Immunohistochemical studies suggested that CB1 receptor expression was higher in animals treated with salicylate and glutamate *via* round window application compared to saline-treated control animals suggesting possible involvement in glutamatergic signaling or synaptic repair (Fauser et al., 2005, AROMWM abstract 352). Datasets from subsequent RNAseq experiments in mice appear to corroborate these findings with *Cnr1* transcripts being detectable in both type I and type II SGNs across multiple ages from E15.5 to P30 (Shrestha et al., 2018; Li et al., 2020). Bhatta et al. reported CB1 receptor immunoreactivity in various cochlear cell types including SGNs, OHCs, IHCs, Dieters’ cells, and in the stria vascularis (SVA) of the lateral wall in adult male Wistar rats (Bhatta et al., 2019). Subsequent transcriptomic analyses also suggest *Cnr1* may be expressed in the mouse cochlea lateral wall where it was detected in fibrocytes and intermediate cells by scRNA-seq (Hoa et al., 2020). The first functional evidence of the role of CB1 receptors in hearing came from a study by Toal et al. (2015) in homozygous CB1 receptor knockout mice. Compared to wild-type controls, mice with homozygous deletion of *Cnr1* showed reduced sensitivity/increased thresholds in a behavioral audiogram task for frequency regions above 8 kHz but had improved Gap detection thresholds on noise that was low-passed at 8 kHz. There was no obvious difference in frequency sensitivity between the control and knockouts as indicated by frequency difference limens (Toal et al., 2015). Although this study clearly indicated a role for CB1 receptors in auditory detection and processing, future experiments are clearly warranted to dissect the functions of CB1 receptor mediated signaling in the adult cochlea particularly with regard to the diversity of potential CB1 ligands that have already been characterized. Furthermore, CreER-mediated conditional deletion or overexpression of *Cnr1* can be employed to determine cell-specific functions and to better understand the extent to which CB1 receptor-mediated signaling is required for SVA, HC, SC, or neuronal functions in the cochlea.

Similar to CB1 receptors, CB2 receptors are also widely distributed throughout the cochlea. Immunohistochemical studies in adult rat cochlea revealed expression of CB2 in the SCs including outer and inner pillar cells, and in the HCs, particularly around the presynaptic ribbon area of IHCs, and also in the SGNs (Martín-Saldaña et al., 2016; Ghosh et al., 2018). Consistent with its known pattern of expression in immune cells, scRNA-seq of adult mouse cochlea lateral wall also suggests that in this area of the cochlea, *Cnr2* is primarily expressed in B-cells and macrophages (Hoa et al., 2020). It is conceivable that such widespread distribution of CB2 receptors may be important for normal hearing. Indeed, pharmacological targeting of CB2 receptor activation by the inverse agonist AM630 or knockdown of *Cnr2* by small interfering RNAs in the inner ear results in significant ABR threshold shifts at 8, 16, and 32 kHz suggesting that tonic activation of CB2 receptors is important for normal hearing (Ghosh et al., 2018). One of the conclusions of these studies was that these hearing deficits might be attributable to loss of ribbon synapses. Indeed, such synaptopathy was observed in response to AM630 and *Cnr2* knockdown and could be reversed by pre-treatment with the CB2 selective synthetic agonist JWH-015 (Ghosh et al., 2018).

In addition to CB1 and CB2 receptors, TRPV1, TRPV2, TRPV3, TRPV4, TRPA1, and TRPM8 have all been shown to respond to CB ligands (Muller et al., 2009). All of these, with the exception of TRPM8, have been shown by quantitative real-time PCR and/or by immunostaining to be readily expressed in the cochlea (Muller et al., 2009; Asai et al., 2010 Asai et al., 2010). Immunostaining studies of TRPV1, 2, 3, and 4 suggest they are enriched in HCs and SCs of the organ of Corti and in the SGNs, while TRPV2 and 4 are also prominent in the SVA (Ishibashi et al., 2008, 2009). Genetic knockout studies in mice reveal that loss of TRPV1, 3, or 4 leads to hearing impairments (Zheng et al., 2003; Tabuchi et al., 2005; Wang et al., 2019) suggesting key roles for these receptors, and thereby ECB signaling, in normal hearing function. In addition, the expression of all four of the TRPV channels can be modulated by ototoxic aminoglycosides (Kitahara et al., 2005; Ishibashi et al., 2009). TRPV1 and TRPV4 in particular have been shown to mediate or mitigate the ototoxic effects of these drugs (Karasawa et al., 2008; Lee et al., 2013; Jiang et al., 2019). Similarly, TRPA1 has been shown to be expressed in the cochlea and in HCs in particular. While genetic knockout of TRPA1 suggests it does not play a major role in normal hearing ability, a subsequent study has revealed that TRPA1 activation facilitates entry of aminoglycosides into cochlear OHCs (Kwan et al., 2006; Stepanyan et al., 2011). Thus TRPA1 represents another possible route by which CB signaling may influence the response to ototoxic insult (Jiang et al., 2019). To summarize, these studies indicate that the ECS is active in the cochlea and contributes to normal hearing function and also to pathological conditions. To further understand the physiological roles of ECB signaling in the auditory periphery, much additional study is likely required. Audiometric functional tests such as ABR and DPOAE must be performed while either inhibiting ECS by pharmacological interventions or in mouse models such germline or conditional knockouts of key receptors or enzymes. Additionally, overexpression models can be brought to bear on these questions. As noted above, conditional models where HC-, neuron-, stria-, or macrophage-specific gene targeting is undertaken can provide greater resolution of cell-specific functions of these molecules.

### ECB Signaling in the Cochlear Nucleus

The cochlear nucleus (CN) serves as the first auditory signal processing hub where information is relayed from the cochlea and then onward along the ascending central auditory pathway. Despite differences with regard to the number of layers of the dorsal CN (DCN) among various species, the laminar DCN can generally be divided into three primary layers: layer 1- the outermost layer or molecular layer, layer 2- the fusiform or pyramidal layer, and layer 3- the polymorphic layer (for reviews, see Brawer et al., 1974; Hackney et al., 1990; Pillsbury, 1996; Oertel and Young, 2004). All the layers consist of various cell types, and generally four types of excitatory glutamatergic projections: those coming from unipolar brush cells, granule cells, and fusiform cells (FC) in layer 2, and those coming from giant cells in the polymorphic layer. Inhibitory inputs originate from either GABAergic interneurons of Golgi cells and stellate cells or, glycinergic interneurons of cartwheel cells (CWC) and tuberculoventral cells (Baizer et al., 2012). The granule cells receive inputs from multiple sources including unmyelinated type II SGNs (Brown et al., 1988), afferent neurons of Scarpa’s ganglion (Burian and Gstoettner, 1988), projections from the octopus cells that detect and encode temporal precision of acoustic stimuli in the VCN, projections from the external cortex of IC (Caicedo and Herbert, 1993) and the auditory cortex (Feliciano et al., 1995). The parallel fibers (PF) from the granule cells send auditory information to the basal dendrites of the FC and also project onto CWCs which in turn send inhibitory inputs to the FCs *via* feed-forward mechanisms. 2AG acts as the primary ECB ligand in the DCN which is synthesized by the activation of either α or β isoform of the DAGL enzyme. Both DAGLα and DAGLβ are expressed in the soma and dendrites of both FCs and CWCs whereas DAGLβ is enriched in the spines of the CWCs and absent in the FC spines. Herkenham et al. first demonstrated very sparse distribution of CB1 receptors in both the DCN and ventral cochlear nucleus (VCN) by radiolabeled ligand binding studies (Herkenham et al., 1991). Later, a detailed quantitative study was performed by Zheng et al. who showed the expression of CB1 receptors in the cytoplasm of different cell types of DCN and VCN including FCs, CWCs, and globular bushy cells in Wistar rats (Zheng et al., 2007). Unique spatial and anatomical distribution of CB1 receptors and DAGL enzymes in the DCN circuit modulate cell-specific short-term plasticity and spike time dependent (STDP) long-term synaptic plasticity (Tzounopoulos et al., 2007; Zhao et al., 2009). Electron microscopy using immunogold labeling revealed that CB1 receptors are distributed in the presynaptic terminals of the PFs that synapse on the FCs and CWCs (Tzounopoulos et al., 2007). Unlike what has been reported in various other parts of the CNS, in the DCN, a higher density of CB1 receptor was observed in the excitatory glutamatergic terminals compared to inhibitory glycinergic terminals. Consistent with this, postsynaptic depolarization caused modulation of excitatory, but not inhibitory, inputs (Zhao et al., 2009). Thus, preferential DAGL expression in CWCs and the resulting CB1 receptor signaling in their presynaptic glutamatergic terminals likely results in suppression of the CWCs. This in turn causes reduced inhibition/increased excitability of the FCs (Zhao et al., 2009). Application of CB1 receptor antagonist AM251 did not affect the basal synaptic transmission in CWCs, suggesting there is no tonic ECB signaling present in these cells. The strength of retrograde ECB signaling can be determined by its ability to briefly reduce synaptic strength of excitatory (DSE) or inhibitory (DSI) inputs. In response to excitatory inputs from parallel fibers, CWCs in the DCN fire rapidly which leads to feedforward inhibition of FCs. Either depolarization or the pairing of an excitatory postsynaptic potential (EPSP) with an action potential (AP) can evoke ECB signaling which preferentially induces DSE at the parallel fiber synapses with CWCs vs. FCs which eventually shifts the balance towards the excitatory output. It is hypothesized that this ECS-mediated lessening of feedforward inhibition may provide ample time for the fusiform cells to incorporate incoming excitatory signals and alter synaptic plasticity by attuning spike time (Zhao et al., 2009). Interestingly, the auditory nerve synapses in the FCs of the DCN are devoid of CB1 receptors and therefore, postsynaptic depolarization of FCs to 10 mV for 1 s did not induce any DSE, suggesting no ECS dependent synaptic strength modulation occurs at the auditory nerve-FC synapses (Zhao et al., 2011). Taken together, the existing literature suggests that components of ECS are readily expressed throughout the CN and can regulate short-term and long-term synaptic plasticity in certain cells and under certain conditions.

### ECB Signaling in the Superior Olivary Complex (SOC), Inferior Colliculus (IC), and Auditory Cortex (AC)

Binaural auditory information first converges in the SOC, which is comprised of three nuclei- the lateral superior olive (LSO), the medial superior olive (MSO), and the medial nucleus of the trapezoid body (MNTB). Excitatory projections from CN project onto LSO of the ipsilateral side, onto MNTB on the contralateral side, and onto MSO bilaterally. Inhibitory glycinergic neurons from MNTB project onto ipsilateral MSO (Grothe et al., 2010). ECB signaling plays a critical role in regulating GABA- and Glycinergic neurotransmission at the MNTB-LSO synapse. Age-dependent expression of CB1 receptors has been reported in the MNTB-LSO circuit. Prior to hearing onset, presynaptic CB1 receptors are ubiquitously found in the MNTB synapses on LSO, MNTB cell bodies, and axons (Chi and Kandler, 2012). The calyx of held is a specialized excitatory glutamatergic synaptic terminal of the axons of bushy cells that project onto inhibitory glycinergic MNTB principal cells (Joris and Trussell, 2018). CB1 receptors are expressed both in the developing soma and calyx in the premature brain, however, CB1 expression becomes restricted to the calyx after hearing onset (Kushmerick et al., 2004; Chi and Kandler, 2012). A tonotopic axis-dependent gradient of CB1 receptor expression is observed in the LSO with lower expression in the high frequency region and higher expression in the low frequency region (Chi and Kandler, 2012). This kind of distribution provides functional benefits in regulating excitatory postsynaptic currents (EPSCs; Chi and Kandler, 2012). The CB1 agonist WIN55,212-2 has been shown to inhibit glutamate release at the calyx and modulate the activity of presynaptic P type Ca^2+^ channels. ECB release in the MNTB is a Ca^2+^-dependent process and ECB released from one synapse can only regulate the activity of the synapse where it was originated, without affecting the neighboring synapses (Kushmerick et al., 2004). CB1 receptors and the endogenous ligand synthesizing enzymes DAGLα/β are also distributed in both glutamatergic and glycinergic terminals in LSO where the ECS functions to balance the release of these excitatory and inhibitory neurotransmitters.

From the SOC, auditory information is next routed *via* the LL to the IC which plays a critical role in auditory processing and also receives afferent inputs from axonal collaterals of the DCN of both ipsilateral and contralateral sides. Both CB1 receptors and CB2 receptors are expressed in the IC and appear to play a role in haloperidol induced catalepsy (Medeiros et al., 2016), however, the function of ECB signaling in acoustic information processing in the IC was not directly explored in this study. In a different study, it was shown that cannabinoid agonists AEA and/or AM251 reduced stimulus-specific adaptation in auditory neurons in the IC of rats (Valdés-Baizabal et al., 2017). Though not a lot to go on, when added to what is known about ECS in the rest of the CNS, it is reasonable to postulate that CB1 (and possibly CB2) receptor signaling can mediate synaptic signaling and plasticity in the IC. However, much more work is needed to more fully elucidate the roles of the ECS in the IC.

The primary auditory cortex is located in the superior temporal gyrus. Immunohistochemical studies in macaques (*Macaca fascicularis*) show distribution of intermediate CB1 receptor immunoreactivity in core auditory cortex areas such as primary auditory cortex A1 and rostral auditory area, and higher CB1 receptor immunoreactivity in the auditory associated belt regions (Eggan and Lewis, 2007). A functional study in mouse cortical slices showed that acute activation of CB1 by the synthetic agonist WIN55,212-2 altered GABA-mediated postsynaptic currents, thus regulating synaptic transmission in the layer 2/3 pyramidal cells in the auditory cortex by inhibiting suppression (Trettel and Levine, 2002). Cannabinoid-mediated influences on suppression were also observed in humans. In a fMRI-based human subject study, Winton-Brown et al. (2011) illustrated that a single acute dose of THC and CBD can modulate neural processing in this area (Winton-Brown et al., 2011). THC administration decreased activation in both primary and secondary auditory regions compared to control and this attenuation of the neural signal was associated with psychosis induction. Unlike THC, another phytocannabinoid, CBD, enhanced activation in the right temporal cortex during auditory processing. Thus, these data suggest that THC and CBD may have opposing effects in auditory stimuli processing which could be due to the fact that CBD can act as a negative allosteric modulator of CB1 receptor (Laprairie et al., 2015). Given the differential responses to these two phytocannabinoids, it is reasonable to speculate that there may also be the complexity of GABAergic responses to ECS and therefore much more careful study is likely required.

Combined, these studies demonstrate widespread expression of ECS components throughout the auditory brain areas. ECB-mediated retrograde signaling regulates both excitatory and inhibitory signaling, thus regulating synaptic signaling and plasticity. Specifically, ECS affects depolarization dependent suppression of excitation (DSE) in the DCN, MNTB, and LSO (Kushmerick et al., 2004; Zhao and Tzounopoulos, 2011; Trattner et al., 2013), and spatially restricted ECS appears critical for sound localization (Trattner et al., 2013). However, further studies are clearly needed to gain higher resolution information regarding the anatomical distribution of ECS components in auditory CNS areas and their roles in hearing.

## Cannabinoids in Auditory Dysfunction and Tinnitus

### Congenital Hearing Loss Associated With ECS Related Genes

ABHD12 catalyzes the hydrolysis of 2AG, which acts as a primary ligand for both CB1 and CB2 receptors. Loss of function mutations of *ABHD12* in humans, and homozygous knockout of this gene in mice, cause polyneuropathy, hearing loss, ataxia, retinitis pigmentosa, and cataract (PHARC) syndrome, which is characterized by early onset of the listed symptoms which notably include hearing loss (Fiskerstrand et al., 2010; Blankman et al., 2013). An *Abhd12* knockout mouse model has revealed that the absence of ABHD12 increases the levels of lysophosphatidylserine in the brain resulting in inflammation (Ogasawara et al., 2018; Leishman et al., 2019) and escalation of microglial activation in the brain in an age-dependent manner (Blankman et al., 2013). These mice also exhibit reduced acoustic startle responses and increased ABR latencies compared to wild type counterparts, though it is unclear the extent to which hearing sensitivity is affected since auditory sensitivity was not tested below 65 dB in the original study (Blankman et al., 2013). Interestingly pharmacological inhibition of ABHD12 did not result in hearing loss in mice, suggesting PHARC-like phenotypes could be associated with *Abhd12* functions in development, as appears to be the case in humans with *ABHD12* mutations (Fiskerstrand et al., 2010). Analysis of *Abhd12* expression based on transcriptomic datasets in the umgear.org database shows that at E16.5, vestibular epithelial cells have higher expression of *Abhd12* compared to the cochlear sensory epithelia. Also, in early postnatal stages (P0 and P1) *Abhd12* is expressed in both sensory hair cells and non-sensory SCs, and in both cochlea and utricle, albeit at higher levels in the utricular SCs (Cai et al., 2015; Kolla et al., 2020). In the adult cochlea *Abhd12* is expressed in the lateral wall, in sensory HCs, and in SCs, though the highest expression appears to be in the IHCs (Liu et al., 2018; Korrapati et al., 2019). In the SGNs, expression of *Abhd12* begins as early as E15.5 and steadily increases until P30 (Li et al., 2020). These expression patterns suggest that some of the pathologies for loss of *Abhd12* function may reside in the cochlea and not just in the CNS. Indeed, an *Abhd12* mutant zebrafish model suggests that loss of *Abhd12* function results in the development of fewer numbers of neuromasts and reduced numbers of hair cells per neuromast (Tingaud-Sequeira et al., 2017). Both mouse and zebrafish models, as well as PHARC symptomology in humans, suggest that demyelination is a primary factor in the observed neurodegeneration which also suggests that myelination of SGNs and other neurons throughout the auditory system might be affected by the loss of ABHD12 function. Indeed, *Abhd12* transcripts are also found in the auditory brainstem including medial geniculate and primary auditory cortex (A1; Guo et al., 2016), and demyelination of neurons in the CNS structures of the auditory system could explain the increased latencies noted in the ABRs of the *Abhd12* knockout mice (Blankman et al., 2013).

To date, there is scant evidence as to whether mutations in other ECS-related genes can cause congenital hearing loss. However, loss of function mutations in ECS-related genes appear to be rare and can cause other significant neurological and neurodevelopmental defects (Smith et al., 2017) which suggests that severe loss of function of some ECS components may be embryonically lethal. Alternatively, loss of function mutations may exhibit only minor or slowly progressive decreases in hearing function and thereby go unnoticed or undiagnosed.

### ECB Signaling in Central Auditory Processing Deficits and Tinnitus

Active ECB signaling in the central auditory pathway has been implicated in regulating selective attention tasks and auditory modulation pathogenesis in schizophrenia. Schizophrenia patients showed larger peak 1 amplitudes while responding to speech sounds compared to healthy subjects, indicating increased auditory nerve activation in the patients (Mathalon et al., 2004). Relatedly, brain event-related potential recordings in cannabis users showed that chronic cannabis use decreases the ability to discern the location, duration, and frequency of a specific tone (Kempel et al., 2003), also suggesting deficits in central auditory processing. This type of altered sensory modulation can be frequently observed in chronic cannabis users who are simultaneously suffering from schizophrenia (Hajós et al., 2008). Related studies in rats suggest that auditory sensory gating can be disrupted by CB1 selective agonists WIN55,212-2 or CP-55940 with possible involvement of the hippocampus, medial prefrontal cortex, and entorhinal cortex (Dissanayake et al., 2008). Combined, these data indicate that disruption of ECB signaling by schizophrenia, long-term phytocannabinoid use, or the combination of these, may lead to disruption of central auditory processing. While more work is needed to better elucidate the mechanisms and potential involvement of ECB signaling in the auditory symptomology of schizophrenia, the body of evidence for ECS involvement in tinnitus (see below) suggests that ECB signaling may not only be important for auditory sensation, but for perception as well, including perception in the absence of physical stimuli which, in the form of auditory hallucinations, is a known symptom of schizophrenia.

Tinnitus is a pathological condition characterized by the perception of non-speech sounds in the absence of acoustic stimuli. These percepts are often characterized in popular culture as tonal “ringing”, but may not always present in this manner. Tinnitus can be acute or chronic and range from mild to severe with chronic suffering often adversely affecting the quality of life. Primary etiological factors of tinnitus include acoustic trauma, head and neck injuries, ear infections, ototoxicity, or high doses of salicylates. While tinnitus is often caused by these factors which include cochlear HC loss, many cases can also be caused by factors unrelated to HC survival or maybe idiopathic. Subjective tinnitus is thought to arise from hyperactivity of the neurons in certain nuclei in the central auditory pathway such as CN, IC, thalamus, or cortex. As the ECS is present in these areas and is known to regulate several synaptic processes including neurotransmission and neuronal activity, it is reasonable to hypothesize that cannabinoid-induced modulation of synaptic function or plasticity could be involved in tinnitus pathogenesis. Unfortunately, human studies to discern the effect of cannabinoids on tinnitus are limited and not wholly consistent in their findings. One case study reported that the administration of dronabinol, the (−)-*trans* isomer of Δ-9-THC relieved tinnitus associated with elevated intracranial pressure (Raby et al., 2006). Though dronabinol seemed to exert a positive effect in that case a separate study using NHANES cross-health survey data found an opposing action of regular marijuana use which was associated with increased prevalence of tinnitus. However in the latter case dose-response was not correlated with the degree frequency or severity of tinnitus (Qian and Alyono, 2020). Counter to the NHANES study another two-year survey the National Survey on Drug Use and Health collected data from more than 29,000 participants between the ages 35 and 49 and showed no association between marijuana consumption and tinnitus (Han et al., 2010). Given the number of potentially psychoactive phytocannabinoids that have been identified in *Cannabis* plants and the differential effects some of these CB ligands can have it may be difficult to determine the influence of CB signaling in tinnitus without better controlling for separate ligands individually. Considering this and other caveats as well as the mixed outcomes of published studies additional work is needed to more accurately dissect the effects of the different CBs and how they might influence auditory perception and tinnitus in humans.

In laboratory animals tinnitus can be modeled by bolus injections of salicylate (Berger et al., 2017) or by noise trauma. Zheng et al. (2010) showed that subcutaneous injection of CB agonists WIN55,212-2 and CP55,940 could not mitigate tinnitus associated behavior, but rather exacerbated such behavior in rodents. Similarly, a subsequent study by this group reported that subcutaneous administration of a 1:1 mixture of Δ-9-THC and CBD could not attenuate acoustic trauma-induced tinnitus (Zheng et al., 2015). Salicylate administration decreases auditory brainstem-evoked responses and alters cortical alpha-band EEG activity. These aspects of salicylate treatment could be reversed by pre-treatment with arachidonyl-2′-chloroethylamide (ACEA) a selective CB1 full agonist, however, ACEA could not attenuate either salicylate or acoustic trauma-induced tinnitus in guinea pigs (Berger et al., 2017). Interestingly, the number of CB1 positive principal neurons appeared to be reduced in the VCN in response to CB1 agonist treatment and remained unaltered in the DCN compared to control animals. While it is difficult to dissect the reason why CB1 expression was differentially regulated in DCN vs. VCN, it is evident that cannabinoid signaling components can be modulated by certain aspects of these tinnitus models. The current existing evidence from multiple studies therefore suggests that activation of CB1 does not mitigate tinnitus, but rather likely exacerbates both acoustic and salicylate-induced tinnitus. These findings appear consistent with reports cited in “ECB Signaling in the Cochlear Nucleus” section of this review that demonstrate increased excitability in DCN in response to cannabinoid signaling. However, there are other non-auditory neuronal networks such as the limbic circuit, and memory and dorsal attention circuits, that can influence tinnitus outcomes (Husain, 2016; Shahsavarani et al., 2021), and these can also be modulated by activation of the CB system. Therefore, additional research is warranted to understand the effects of exogenous CB agonists on tinnitus or on auditory perception particularly given the relatively widespread use of *Cannabis* products and the growing incidences of their use as therapeutics for a variety of conditions including epilepsy and pain management.

### Cannabinoids and Otoprotection in the Cochlea

CB2 receptors are ubiquitously expressed in immune cells and CB2-mediated signaling is considered to be the primary component of the ECS in regulating immune responses. 2AG and AEA binding to CB2 receptors regulates the release of inflammatory cytokines and has been implicated in preventing neuronal damage by suppressing inflammation in neurodegenerative diseases and autoimmune disorders (Achiron et al., 2000; Pryce et al., 2003; Carrier et al., 2004). Activation of CB1 receptors in the CNS can also mitigate inflammation and offer neuroprotection in neurodegenerative diseases like multiple sclerosis (Pryce et al., 2003). CB1 receptors are also expressed along with CB2 in some immune cells such as macrophages (Han et al., 2009; Mai et al., 2015), mast cells (Facci et al., 1995), and dendritic cells (Svensson et al., 2010) and can participate in immunomodulation. ECB ligands also bind to capsaicin-sensitive TRPV1 channels which can modulate immune responses in rheumatoid arthritis (Lowin and Straub, 2015), hyperalgesia and chronic pain (Maione et al., 2006), and gastrointestinal function (Acharya et al., 2017). Not much is known about immunomodulation in the auditory system, though more and more evidence are emerging regarding the roles of immune cells and cytokine signaling in cochlear development and otoprotection (Hu et al., 2018). Thus, herein, the discussion of ECB signaling will be on otoprotection, though readers are referred to Pandey et al. (2009) for further information on ECB signaling in immune function.

Emerging evidence in the past decade has shown that resident immune cells are essential for cochlear function and immune surveillance to maintain homeostasis and function in the cochlea. Bone marrow-derived macrophages populate the cochlea in the adult stage, and these resident macrophages respond to cochlear insults *via* fractalkine receptor CX3CR1 mediated signaling (Okano et al., 2008; Hirose et al., 2017). These cells are primarily distributed in the auditory nerve and lateral wall, though smaller numbers of macrophages have been noted in the spiral limbus, inner and outer sulci, and surrounding the basilar membrane. During homeostatic conditions, the OC is largely devoid of macrophages. However, in response to damage mediated by various otototoxic insults including noise, trauma, and ototoxic drugs, reactive oxygen species (ROS) are produced. The ROS production in turn leads to upregulation of pro-inflammatory cytokines such as TNF-α, COX2, and multiple interleukins, including IL-1β and IL-6, in the OC, lateral wall, and SGNs (Fujioka et al., 2006; So et al., 2007; Huth et al., 2011; Hwang et al., 2011; Ghosh et al., 2018). In turn, this cytokine signaling recruits macrophages to these areas of the cochlea, including the OC. During the initial phase of the immune response, CX3CR1+ resident macrophages are activated to ameliorate inflammation and remove cellular debris (Gregory and Devitt, 2004). However, sustained immune activation results in apoptosis of HCs and the apoptotic cells release CX3Cl1 which signals the systemic CX3CR1+ CD45+ macrophages and monocytes to infiltrate the cochlea which can then cause further damage to HCs and SGNs (Kaur et al., 2015). Activation of CB signaling by the endogenous ligands, AEA or 2AG, or by synthetic agonists such as HU-308, CP55,940, or WIN55,212-2, attenuates such pro-inflammatory cytokine production in various inflammatory pathological conditions like lipopolysaccharide (LPS)-mediated inflammation, arthritis, ischemia, and reperfusion injuries (Cabral et al., 1995; Berdyshev et al., 1997; Rajesh et al., 2007; Selvi et al., 2008). Indeed, it has been shown that activation of CB2 signaling by the synthetic cannabinoid JWH-015 mitigated cisplatin-induced pro-inflammatory cytokine production in the rat cochlea and exerted otoprotective effects (Ghosh et al., 2018). Specifically, activation of CB2 receptors by local administration of JWH-015 protected against cisplatin-induced hearing loss in male Wistar rats by mitigating inflammation, preventing cisplatin-mediated loss of ribbon synapses and limiting OHC death *via* inhibition of ERK/MAPK signaling (Ghosh et al., 2018).

Moreover, cisplatin treatment and other ototoxic insults can activate factors such as ERK and NF-κB, which in turn increase the production of pro-inflammatory cytokines like TNF-α, IL-1β, and IL-6 (So et al., 2007). Though not yet directly shown in the ear, these cytokines can be inhibited in other systems *via* activation of the CB2 receptor (Liu et al., 2014). Activation of TRPV1 and CB2 receptors by capsaicin and/or AEA has been implicated in the regulation of macrophage infiltration in the gut (Acharya et al., 2017), and reduced macrophage chemotaxis in mice (Sacerdote et al., 2000, 2005). In the brain, CB receptors cannot be detected in resting microglia, but their expression is upregulated in pathological conditions like multiple sclerosis and Alzheimer’s disease. This upregulation can be therapeutically targeted to mitigate inflammation (Stella, 2010). Future studies in knockout mouse models or by pharmacological modulation of CB signaling components, can therefore further unravel the role of CB signaling in cochlear inflammation. Understanding the function of different components of the ECS is therefore likely critical for designing therapeutic targets to ameliorate cochlear inflammation.

In addition to CB receptors, transient receptor potential (TRP) channels have also been shown to be bound and activated by CB ligands. In fact, the first endogenous ligand to be discovered for TRPV1 was AEA (Muller et al., 2009). Similar to the canonical CB receptors, TRPV1 is also thoroughly distributed throughout the cochlea, being detected in the apical membranes of hair cells, neighboring SCs, and in the stria vascularis (Zheng et al., 2003; Mukherjea et al., 2011; Jiang et al., 2019). Capsaicin, a component of hot chili pepper and known activator of TRPV1 has been shown to protect against cisplatin-mediated hearing loss by mitigating OHC cell death (Bhatta et al., 2019). Intriguingly, this otoprotective effect was at least partly mediated by CB2 signaling suggesting that ligands such as capsaicin which can be targeted to both CB2 receptors and TRPV1 may provide otoprotection *via* multiple mechanisms. Though, it has been suggested by multiple studies that TRPV1 activation can actually exacerbate cisplatin-induced ototoxicity (Mukherjea et al., 2008; Di et al., 2020). Thus, while it is possible that otoprotective effects of capsaicin or other CB-like ligands may be mediated by CB2 receptors rather than TRPV1, it is also possible that TRPV1 may have differential responses to different modes of activation. Indeed, while both cisplatin and capsaicin can activate TRPV1, capsaicin appears to elicit different downstream effects, tilting the balance between pro-apoptotic Ser^727^ phosphorylation of Signal transducer and activator of transcription 1 (STAT1) and anti-apoptotic Tyr^705^ phosphorylation of STAT3 towards cell survival (Bhatta et al., 2019). Conversely, systemic administration of cisplatin or bacterial LPS causes upregulation of TRPV1 and STAT1 expression in the cochlea, but does not result in altered phosphorylation states of STAT1 and STAT3 in the same manner as capsaicin. Thus cisplatin activation of TRPV1 induces inflammation, apoptosis, and hearing loss (Mukherjea et al., 2008, 2011; Ghosh et al., 2018; Jiang et al., 2019), but capsaicin or other CB ligands might activate TRPV1 in ways that are otoprotective. Furthermore, cisplatin-mediated TRPV1 upregulation can be blocked by transtympanic administration of the CB2 selective agonist JWH-015 suggesting potential feedback mechanisms that may limit the negative effects of TRPV1 on hair cell survival (Ghosh et al., 2018). As several reports have suggested that TRPV1 activation, in a manner similar to cisplatin, may also mediate aminoglycoside uptake and toxicity in the cochlea (Jiang et al., 2019) it is reasonable to speculate that CB ligands may exert protective effects under these conditions as well. However, this has yet to be directly tested. In summary, there are currently only a few direct studies that demonstrate otoprotective effects of ECS or exogenous cannabinoids; however, these early results are promising and suggest that CBs may have potential as therapeutic agents for the prevention of HC loss.

## ECB Signaling in The Development of The Auditory System

Currently, there is limited information regarding cannabinoid signaling during inner ear development. However, with the recent publication of several different transcriptomic studies that have profiled the developing inner ear it is possible to arrive at a rudimentary description of the expression and distribution of ECS components. Furthermore, given what is known about ECB signaling in the development of other parts of the nervous system, it is possible to provide some insight into the potential roles the ECS may play during otic development.

Specifically, research from the brains of both mice and rats suggest that ECS components are expressed fairly early in terms of neurological development and contribute to the regulation of neural stem cell and progenitor proliferation, glial and neuronal differentiation, neurite formation, axonal growth and pathfinding, and synaptic connectivity (Galve-Roperh et al., 2006). In support of the critical involvement of ECS in neural development, studies of rat brain tissues suggest that 2AG expression is highest during late embryological development, and perinatally, declining thereafter into adulthood (Berrendero et al., 1999). This expression of 2AG is mirrored by its precursor molecules and its degradation enzymes. The lipases sn1-DAGLα/β, which are necessary for the synthesis of 2AG, are expressed as early as E10 in mouse axonal tracts and E14.5 in long-range telencephalic axons. At E18.5 the expression becomes localized to postsynaptic dendrites of glutamatergic pyramidal cells, where it likely influences axonal growth cone pathfinding of presynaptic cells. Indeed, it has been shown in neonatal rodent models that CB1 receptor signaling induced by 2AG is critical for axonal growth and pathfinding (Williams et al., 2003; Berghuis et al., 2005). This includes activation of CB1 in cerebellar neuron cultures where treatment with agonists promoted neurite outgrowth in a dose-dependent manner (Williams et al., 2003). Furthermore, this process could be inhibited by blocking DAGLα/β activity by tetrahydrolipstatin (Bisogno et al., 2003). Additionally, (endo) cannabinoid signaling can negatively influence neurite extension in the CNS in some cases by inhibiting TrkB signaling (Berghuis et al., 2005). In retinal ganglion cells, ECB signaling can modulate growth cone organization by interacting with the proteins netrin and deleted in colorectal cancer (DCC) (Argaw et al., 2011; Duff et al., 2013). Similarly, activation of cannabinoid signaling by 2AG promotes Slit2 and Robo1 accumulation in oligodendrocyte end-feet and axonal growth cones respectively and modulates steering of axonal growth direction in the forebrain (Alpár et al., 2014). In addition to these roles in axon elongation and pathfinding, CB signaling has been shown to promote neurosensory progenitor cell proliferation and neurogenesis in both the embryonic and adult hippocampus (Jiang et al., 2005). Cortical neurogenesis appears to be similarly regulated by CB signaling which again has been shown to promote the generation and maturation of new neurons and to influence cell fate decisions between deep layer or upper layer neurons (Paraíso-Luna et al., 2020). CB signaling is also important in glial development, in particular promoting the differentiation of oligodendrocytes and subsequent levels of myelination (Huerga-Gómez et al., 2021). As similar processes, including oligodendrogliogenesis, neurogenesis, axonal pathfinding, and synaptic innervation, occur during the development of the cochlea (Webber and Raz, 2006) it is suggestive that ECB signaling may be critical for the establishment and function of the inner ear.

### Expression of ECS Components in the Developing Inner Ear

The sensory structures of the inner ear first develop from a placodal thickening of the lateral ectoderm (the otic placode) that subsequently invaginates to form the otic cup which is then fully internalized to form the otic vesicle. The vesicle then elongates, undergoing morphogenesis that ultimately leads to the formation of the endolymphatic sac, the spiral-shaped cochlear duct, the vestibule, the semicircular canals, and the nonsensory and sensory tissues of the auditory and vestibular periphery. In mice, the otic placode forms around embryonic day (E) 8.5, the otic cup around E9, and the vesicle around E9.5. Subsequently, the vestibular tissues begin to develop (becoming established between E12.5 and E13.5) and the cochlear epithelium also begins to develop and differentiate with hair cells appearing between E13.5 and E15. Both auditory and vestibular end-organs subsequently continue their maturation beyond gestation and into the first two postnatal weeks. Hearing onset in mice occurs around postnatal days (P) 9–14 generally becoming fully mature by around 3–4 weeks of age. Herein we examined gene expression patterns of ECS receptors and metabolic enzymes from four studies. Hartman et al. (2015) utilized a >19,000 unique gene microarray to profile gene expression from microdissected otic vesicles, periotic tissues, and the remainder of the embyros of E10.5 mice. Muthu et al. (2019) performed RNA-seq on otic vesicles derived from E11.5 mouse embryos. Kolla et al. (2020) performed single cell RNA-seq from murine cochlear epithelia isolated at E14, E16, P1, and P7. Finally, Li et al. (2020) sorted SGNs from mouse embryos at E15.5 and from postnatal mice at P1, P8, P14, and P30, while also isolating glial cells from the inner ear at P8.

**Figure 5:**
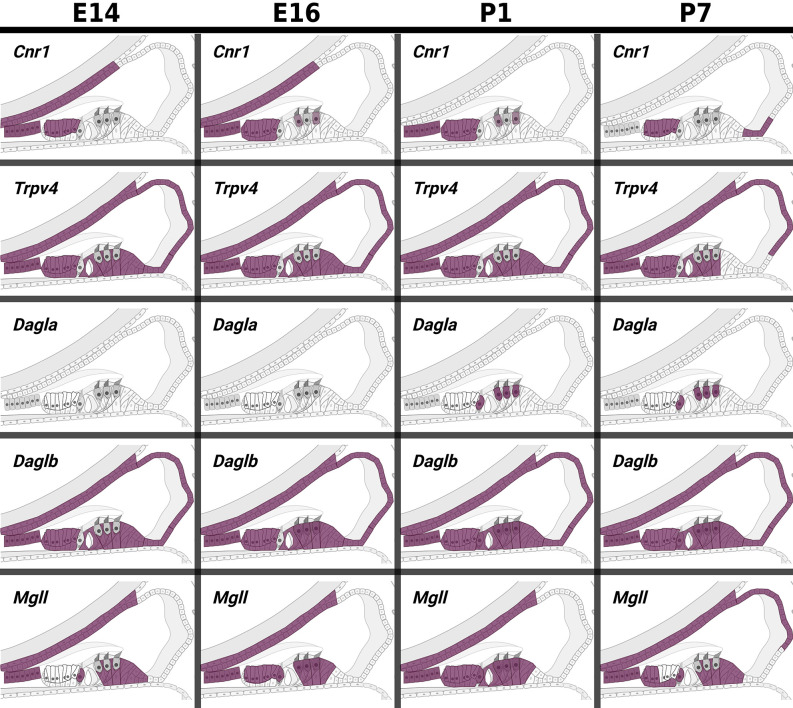
*In pseudo* hue-visualization of the patterns of expression of ECS components in late embryonic and early postnatal cochlear duct. Using data from Kolla et al. (2020); the distribution of several ECS component transcripts are plotted onto a schematic of the mammalian cochlear duct. Purple color indicates areas where transcripts are likely to be detected based on bioinformatic grouping of scRNA-seq data from mouse cochlear epithelia at embryonic days E14 and E16, and postnatal days P1 and P7.

Data from experiments conducted using E10.5 and E11.5 otic vesicles (Hartman et al., 2015; Muthu et al., 2019) suggest that several of the ECS components are not detectable or are expressed at very low levels. However, *Cnr1*, *Trpv4*, *Napepld*, *Daglb*, *Faah*, and *Abhd12* do appear to be expressed at physiologically relevant levels (Tables s 1, 2). In particular, *Faah* and *Abhd12* demonstrated fairly robust levels of expression at E11.5 (Table 2). As these two transcripts encode for enzymes that degrade CB ligands, the general trend in the data suggest that ECB signaling is not highly active and perhaps even specifically inhibited during OV development at E10.5–E11.5. Looking at cells remaining within the cochlear duct from E14 onward (i.e., not neurons that have delaminated or their surrounding glia), it is clear that a number of ECS components are expressed throughout the cochlear epithelium (Figure 5). Most notably, *Cnr1* appears to be expressed in the medial portion of the cochlear duct (in interdental cells, the greater epithelial ridge (GER), and *Oc90* positive cells), and *Trpv4* appears to be expressed everywhere except the hair cells. Aside from these, expression of the rest of the receptors (*Cnr2* and the other *Trpv* transcripts) appears low or undetectable. With regard to CB enzymes, *Dagla* appears to be expressed in both IHCs and OHCs postnatally (P1 and P7), but is low to undetectable at embryonic stages. In contrast, *Daglb*, appears widely distributed from E14–P7, though it is also largely absent from the HCs at embryonic stages. *Mgll* appears to be fairly widely distributed from E14–P1 and is then largely downregulated in PCs and the lateral GER, though it is interesting that *Mgll* was not readily detected in OHCs at any of the time points. *Abhd6* and *Abhd12* a had fairly widespread distribution from E14 to P7. In general, the levels of expression and the numbers of cells with detectable transcripts could be described as moderate, with none of the components being expressed as highly as transcripts such as *Gata3*, *Sox9*, *Tecta*, or *Hes5* which are known to be highly expressed in the cochlear epithelium. However, the expression levels of ECS components, when present, were consistent with levels that have been observed for other transcripts known to play vital roles in cochlear development like *Lfng, Prox1, Cdh2*, and *Cdh23*.

**Table 1:** Expression of ECS-related transcripts from reported RNA-seq values using E10.5 otic vesicles and surrounding tissues. Data from E10.5 was obtained from Hartman et al. (2015) and are presented as untransformed microarray probe intensities. The last four rows demonstrate values obtained for transcripts known to be highly expressed (Fbxo2, Gata3) or low to undetectable (Atoh1, Cdkn1b) at these stages of otic vesicle (OV) development. Dashes in grayed-out boxes indicate cases where values were not found in the original dataset. It is important to note that since this report utilized a microarray, undetectable values may not represent lack of expression, but possibly lack of a probe and therefore these were not assigned a zero value or included in the heat map.

	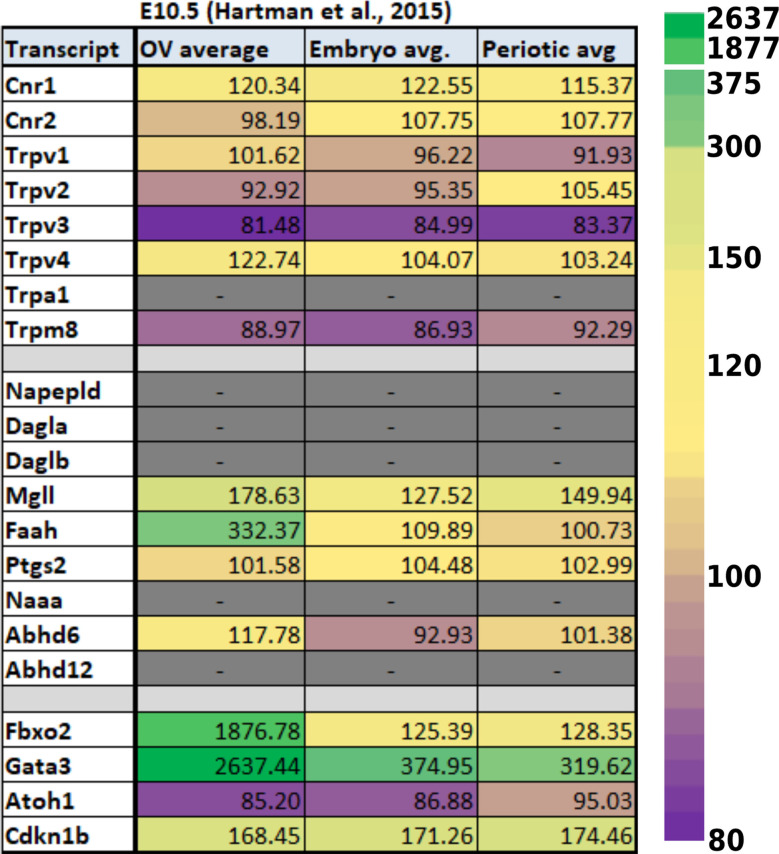

**Table 2:** Expression of ECS-related transcripts from reported RNA-seq values using E11.5 otic vesicles. Data from E11.5 embryos were obtained from Muthu et al. (2019) and is presented as reads per kilobase million (RPKM). The last four rows demonstrate values obtained for transcripts known to be highly expressed (Fbxo2, Gata3) or low to undetectable (Atoh1, Cdkn1b) at these stages of otic vesicle (OV) development.

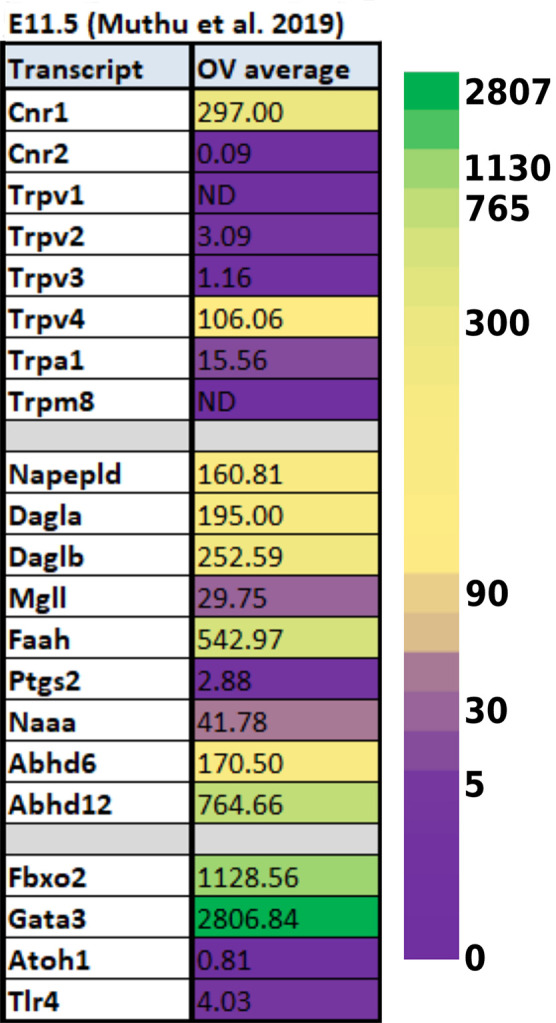

In the developing SGNs, there are also a fair number of ECS components that have been detected through transcriptomic profiling. Some of the components are even expressed at very high levels and in intriguing patterns. For example, *Cnr1* is expressed in E15.5 SGNs at a level of 9,161 counts per million (CPM, Table 3) which is about the same level of expression one finds for the transcript that codes for parvalbumin (*Pvalb*) in mature SGNs and greater than 4-fold higher than the expression level of the glutamate receptor AMPA type subunit 2 (*Gria2*). Interestingly, *Cnr1* expression then appears to steadily decrease in the SGNs by more than 7-fold from E15.5 to P14.

**Table 3:** Expression of ECS-related transcripts in the developing spiral ganglion. Data were obtained from Li et al. (2020) and are presented as counts per million (CPM) mapped reads. Transcripts presented in the bottom five rows are not known to be directly related to ECB signaling, but are presented to provide reference values for transcripts that are expressed in the SGNs at higher (NeuroD1, Gria2, Pvalb) and lower (Sox2, Gjc3) levels, and expressed in glial cells in an inverse manner.

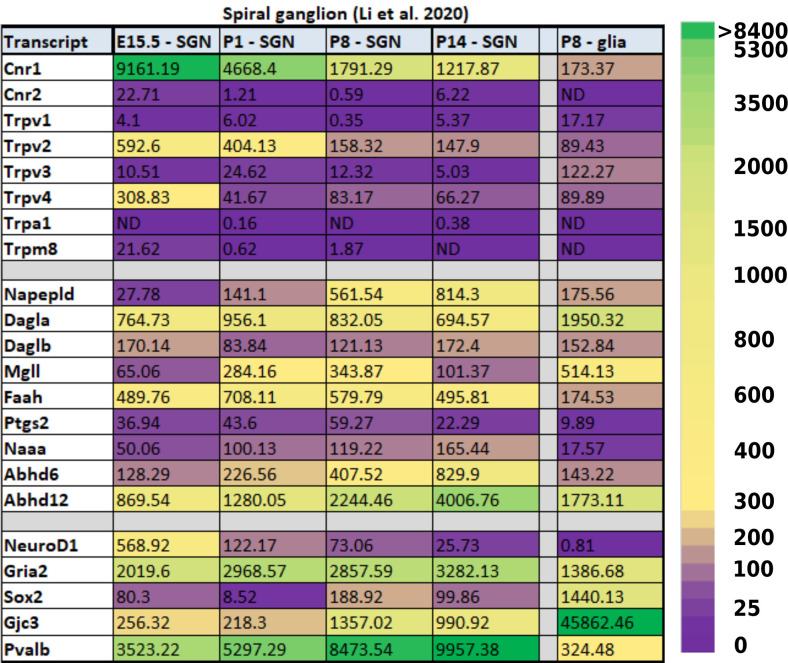

While the level of expression of *Cnr1* at P14 is still certainly high enough for it to continue to exert physiological function (1,218 CPM), the remarkably high expression embryonically and its subsequent downregulation hint that it may be playing a key developmental role. Indeed, the fact that several of the ECS components are so readily detected in the cells and tissues of the developing inner ear suggests that ECS may be playing a vital role in several key developmental processes, most likely the establishment and refinement of the neuronal connections to the HCs (Bisogno et al., 2003).

### Putative Functions of ECB Signaling in Otic Development

During otic vesicle development, both *Dagla* and *Daglb* are expressed at E15.5 and persist throughout hearing onset and maturation in the spiral ganglion. Therefore, it is conceivable that 2AG mediated CB signaling plays a role in radial migration and pathfinding of auditory nerve fibers. The 2AG signaling pathway has been shown to be important for oligodendrocyte progenitor cell proliferation, maturation, and myelination (Gomez et al., 2010). Along these lines, *Dagla* and *Daglb* genes are also expressed in the glial cells at P8, suggesting 2AG mediated signaling in glial cells could regulate oligodendrogliogenesis and myelination, though such a hypothesis would be better founded on expression data from earlier ages and, of course, direct testing of this hypothesis with loss of function experiments.

Transcripts for the receptor *Cnr1* are expressed in the developing SGNs at least as early as embryonic day (E)10.5, but become extremely plentiful at E15.5 and decline thereafter, though are still readily expressed at P14. During this period, the SGNs undergo critical development including significant refinement, functional maturation, and synaptic pruning. Around E15, peripheral axons extend to the cochlear epithelium, indicating CB1 may be involved in axon growth cone formation and migration as it is seen in radially migrating glutamatergic neurons traveling to the cortical preplate, and in migrating interneurons (Saez et al., 2014). In addition to functioning in the migration and connection of the afferent fibers, cannabinoid signaling may also be playing a role presynaptically in the IHCs during this time. Ribbon synapses are specialized electron-dense structures that allow for rapid transmission of information *via* rapid docking and release of multiple neurotransmitter containing vesicles. These specialized presynaptic structures are found in retinal cells and in inner ear hair cells. Mature IHCs, contain one ribbon per active zone, anchored to the membrane, which tethers a single layer of vesicles (Schmitz et al., 2000; Nouvian et al., 2006). The number of ribbon synapses not only varies among the auditory organs of various species, but it also changes during the development and hearing maturation of an organism (Voorn and Vogl, 2020). ECS receptors and enzymes are canonically found in both pre and post-synaptic terminals in the brain and regulate synaptic strength and plasticity (Castillo et al., 2012). However currently, we have rudimentary knowledge on the distribution and function of ECS components in the development or function of cochlear ribbon synapses. However, a recent study in the developing (3-day post-fertilization) zebrafish has shown that *Cnr2* is expressed in both the sensory epithelia (SE) of the inner ear and lateral line neuromast. Loss of function of the *cnr2* gene resulted in the formation of immature synapses and irregular tethering of ribbons as well as changes in size, shape, and number of the neurotransmitter containing vesicles and resulted in accelerated vesicular trafficking (Colon-Cruz et al., 2020). So far, it remains unknown, whether CB2 activation plays similar roles in rodents or other mammals, but the study is suggestive that ECB signaling could play a critical role in the establishment and maintenance of ribbon synapses in cochlear IHCs. In rodents, from embryonic development at E15.5 until hearing onset, numerous numbers of dynamic changes take place in the SGN including axonal branch refinement, axon myelination, and synaptic pruning, that could potentially influence ribbon synapse formation (Coate et al., 2019). Transcriptomic data analysis showed that several major components of canonical CB signaling, e.g., *Cnr1* and *Dagla*, are expressed in the SGN around this time, making it conceivable to hypothesize roles for endocannabinoid signaling in axonal guidance, and synaptic maturation in the cochlea. Additionally, prior to hearing onset, during the first two postnatal weeks of development in mouse cochleae, calcium spikes are generated in the IHCs inducing spontaneous firing of the SGN (Tritsch et al., 2007; Wang and Bergles, 2015). These calcium spikes incite electrical activity in the entire auditory circuit and play a crucial role in maturation and formation of auditory circuits (Kandler et al., 2009; Clause et al., 2014). As described above, retrograde cannabinoid signaling regulates synaptic strength by DSE and DSI at the cochlear nucleus and MNTB-LSO which can modulate the strength of spontaneous activity and possibly modulate the development of MNTB-LSO prior to hearing onset (Chi and Kandler, 2012). Additionally, activation of the ECS through Gq coupled GPCR was shown to increase calcium release in astrocytes in a tripartite synapse, which could induce glutamate release and binding to postsynaptic NMDA receptors (Navarrete and Araque, 2008). Given the fact that ECS components appear to be expressed in developing SGN and cochlear tissues during these stages of development and maturation, it is also plausible to hypothesize that cannabinoid signaling is involved in regulating calcium signaling in the greater epithelial ridge, which needs to be investigated.

While the primary functions of ECS have been thought to be relating only to synaptic and immune function, a growing body of evidence suggests that cannabinoid signaling regulates the survival and proliferation of neural progenitor cells (NPCs) and neural stem cells (NSCs) in a dose-dependent manner (Zorina et al., 2010; Gaffuri et al., 2012; Prenderville et al., 2015). Primarily, CB1/CB2 receptor downstream signaling activates PI3/Akt and ERK to promote NPC proliferation and differentiation (Rueda et al., 2002; Molina-Holgado et al., 2007; Palazuelos et al., 2012; Compagnucci et al., 2013) or regulate the expression of genes that contribute to self-renewal or cell fate decisions by CREB phosphorylation (Isokawa, 2009). Administration of the synthetic CB agonist HU-210 to embryonic mice leads to increased proliferation of cerebellar granular cells. *in vitro* studies suggest that CB1 receptor binding may promote proliferation through the PI3K/AKT/GSK/3β/β-catenin signaling pathway (Trazzi et al., 2010). Cannabinoid receptors can also interact with cytokine signaling pathways such as IL-6/JAK-STAT3 and IL-1β to regulate neurite outgrowth and NSC proliferation, respectively (Zorina et al., 2010; García-Ovejero et al., 2013). These cytokines are implicated in inflammatory immune responses to noise trauma and other pathological conditions such as tinnitus and otitis media (Fujioka et al., 2006; Hwang et al., 2011; MacArthur et al., 2011; Vethanayagam et al., 2016), but could also be important in the generation of SGNs or other neurons in auditory CNS structures. Activation of CB1 by (R)-(+)-Methanandamide enhanced self-renewal of Sox2 positive subventricular zone cells *in vitro* *via* interaction with Notch signaling (Xapelli et al., 2013). Notch signaling plays critical role in the establishment of the inner ear sensory epithelia and in cell fate decisions during later inner ear development (Brown and Groves, 2020). Thus, it is enticing to speculate that ECS may play a role in the development of the organ of Corti, or of HCs or SCs. However, studies that directly probe these putative roles for ECS in cochlear development are currently lacking. Also, the relatively lower levels of expression of ECS components at E10.5 and E11.5 when much of the proliferation and differentiation of neuronal and sensory epithelial precursors is occurring suggests that perhaps ECB signaling is not as critical in these processes as it is during later stages of development. However, there is currently limited data that could illuminate the distribution or expression of ECS factors between E11 and E14 in mice. This makes it difficult to assess the extent to which ECB signaling may play a role in the formation of the spiral ganglion and otic vesicle as much of the delamination of neuroblasts from the OV takes place during this time and proliferation of neural cells contributing to the SGN ceases around E14.5 (Fritzsch et al., 2015). Similarly, this window is thought to be critical for hair cell and supporting cell differentiation. Future studies are therefore warranted to more fully elucidate whether CB signaling plays a role in neurogenesis or in hair cell development in the inner ear.

## Discussion

Here we review what is currently known about the distribution of endocannabinoid components including the receptors and metabolizing enzymes in the auditory circuit based on immunohistochemical evidence, transcriptomic data, and functional studies present in the literature. Cannabinoid receptors CB1, CB2, and non-canonical cannabinoid receptors of the TRP channel family are expressed throughout the peripheral and central structures of the auditory system. In the central auditory system, retrograde cannabinoid signaling regulates synaptic plasticity and synaptic strength by modulating both excitatory and inhibitory synapses. However, very little is known about the function of the ECS in the auditory periphery particularly with regard to developmental studies. Recent reports suggest that an endogenous cannabinoid tone may be present in the cochlea which is required for normal hearing. Furthermore, recent work suggests that boosting of this endogenous system can protect against ototoxic insults like cisplatin by ameliorating inflammation and inducing anti-apoptotic signals that prevent OHC death. While a number of studies in humans and in animal models suggest many beneficial aspects of ECS signaling or the potentiation thereof for hearing function, there does seem to be an exception to this in the case of tinnitus or other cases where auditory hypersensitivity or hallucinations may occur. Indeed, while there may be complexities yet to be resolved regarding cannabinoid signaling and tinnitus, so far, the results suggest that CBs may worsen tinnitus, or, at best, have no effect.

Of particular note and focus for this review, it is important to highlight more recent findings that demonstrate how the endocannabinoid system can influence neuronal cell growth, proliferation, and differentiation in the CNS. This adds significantly to the established understanding that ECS components are also localized in the axon growth cones and regulate axon guidance and trafficking as well as synaptogenesis and synaptic function. However, currently, the role of cannabinoid signaling in the auditory system is not well explored, particularly with regard to traditional functions in axonal pathfinding and establishment and maintenance of synapses in the spiral ganglion, but also with regard to possible roles in the proliferation or differentiation of auditory sensory cells, supporting cells, neurons, or oligodendrocytes. Transcriptomic data reveals that multiple components of the ECS are present in the developing otic vesicle at least as early as E10.5 and the expression of many ECS components persists until hearing matures. Loss of function mutations in ECS genes in zebrafish, mice, and human populations all suggest critical developmental roles, including important functions in the establishment and innervation of sensory epithelia and therefore it is critical that future studies more closely examine the role of ECS in these processes and in otic development and function.

## Author Contributions

SG and BW conceived the project. SG, KS, and BW wrote and edited the manuscript. SG, KS, and BW created figures and tables. All authors contributed to the article and approved the submitted version.

## Conflict of Interest

The authors declare that the research was conducted in the absence of any commercial or financial relationships that could be construed as a potential conflict of interest.
